# Global Trends and Current Status in Osteonecrosis of the Femoral Head: A Bibliometric Analysis of Publications in the Last 30 Years

**DOI:** 10.3389/fendo.2022.897439

**Published:** 2022-06-15

**Authors:** Zeqin Wen, Yusheng Li, Zijun Cai, Meng Fan, Jian Wang, Ran Ding, Cheng Huang, Wenfeng Xiao

**Affiliations:** ^1^ Department of Orthopaedics, Xiangya Hospital, Central South University, Changsha, China; ^2^ National Clinical Research Center for Geriatric Disorders, Xiangya Hospital, Central South University, Changsha, China; ^3^ Department of Orthopedics, China-Japan Friendship Hospital, Beijing, China

**Keywords:** osteonecrosis of the femoral head, bibliometric analysis, global trends, current status, the last 30 years

## Abstract

**Introduction:**

Osteonecrosis of the femoral head (ONFH) is a progressive and disabling disease with severe socioeconomic burdens. In the last 30 years, a growing number of publications have reported significant advances in understanding ONFH. However, only a few studies have clarified its global trends and current status. Thus, the purpose of our study was to summarize the global trends and current status in ONFH through bibliometrics.

**Materials and Methods:**

Publications related to ONFH from 1991 to 2020 were searched from the Web of Science (WOS) core collection database. The data were analyzed with bibliometric methods. Microsoft Excel was used for statistical analysis and to draw bar charts. SPSS was applied to perform linear regression analysis. VOSviewer was used to conduct bibliographic coupling analysis, co-authorship analysis, co-citation analysis and co-occurrence analysis.

**Results:**

A total of 5,523 publications were covered. The United States consistently ranked first in total publications, sum of times cited, average citations per item and H-index. Kyushu University was the main contributor to ONFH. *Clinical Orthopaedics and Related Research* was the major publishing channels for ONFH-related articles. Takuaki Yamamoto published the most ONFH-related articles. Studies regarding ONFH could be divided into five clusters: 1) mechanism study, 2) treatment study, 3) complication study, 4) radiological study and 5) etiological study. Mechanism study might become a hot spot in the future.

**Conclusions:**

The total number of publications in ONFH has generally increased over the last three decades. The United States was the leading country in ONFH research. Transplantation, engineering, cell and molecular biology, pharmacology and endocrinology have gradually increased and become hot topics in ONFH research. Mechanism study in ONFH including mesenchymal stem cells, apoptosis, oxidative stress, adipogenesis, osteogenic differentiation and endothelial progenitor cells, have attracted more attention and will become a hot spot in the future.

## Introduction

Osteonecrosis of the femoral head (ONFH) is a disease in which local death of osteocytes and bone marrow components occurs due to venous stasis or impaired arterial blood supply or disruption of the femoral head (1). ONFH can be divided into two major types: traumatic and nontraumatic ONFH (1). Traumatic ONFH is mainly caused by femoral head and neck fracture, acetabular fracture, hip dislocation, and severe hip sprain or contusion (2–5). The main etiology for nontraumatic ONFH is long-term or excessive glucocorticoid (GC) administration, alcohol overconsumption, hemoglobin diseases and autoimmune diseases (1, 6, 7). However, the detailed pathogenesis of ONFH is not fully understood (8). Severe ONFH can lead to the collapse of subchondral bone and eventually damage to the hip joint, resulting in labor capacity losses for themselves and substantial economic losses for their families (9). It is estimated that 20,000 new cases of osteonecrosis are diagnosed in the United States each year, and the cumulative number of patients with ONFH ranges from 300,000 to 600,000 (10). Patients with advanced ONFH often do not respond well to medical treatment and require total hip arthroplasty (THA). However, THA, especially for young people, may lead to a series of complications, including dislocation, periprosthetic fracture, infection and prosthesis loosening (11). Therefore, the severe socioeconomic burdens and limited treatment options have forced us to continue to study the pathogenesis of ONFH and develop effective treatments for ONFH.

In the last 30 years, a growing number of publications have reported significant advances in the pathogenesis and treatment of ONFH worldwide. However, to the best of our knowledge, only a few studies have clarified the global trends and current status in ONFH. Bibliometrics is a method to cognize the global trends and current status of a certain field and evaluate the contributions of a collection of research results such as all publications of the same scholar, institution or country, by collecting the metrology characteristics of the publications (12–14). In addition, bibliometrics can also be used to guide policy formulation (15). Currently, bibliometric analysis has been used in a wide range of fields, including anesthesia, cancer, orthopedics and neurology, to compare the contributions of different research findings (13, 16–18). Therefore, the purpose of our study is to evaluate and summarize the global trends and current status in ONFH by analyzing ONFH-related publications in the last 30 years with bibliometrics to help researchers understand the research perspectives, hot spots and trends of ONFH.

## Materials and Methods

### Data Sources

The search was conducted using the Web of Science (WOS) Core Collection database, including Science Citation Index Expanded (SCI-Expanded), Social Sciences Citation Index (SSCI), Arts and Humanities Citation Index (A&HCI), Conference Proceedings Citation Index-Science (CPCI-S), Conference Proceedings Citation Index-Social Science and Humanities (CPCI-SSH), Book Citation Index-Science (BKCI-S), Book Citation Index-Social Science & Humanities (BKCI-SSH), Emerging Sources Citation Index (ESCI), Current Chemical Reactions Expanded (CCR-Expanded) and Index Chemicus (IC). The journal impact factors (IF) came from the 2020 version Journal Citation Reports except for *Journal of Bone and Joint Surgery-British Volume* from the 2014 edition and *Proceedings of the Institution of Mechanical Engineers. Part H-Journal of Engineering in Medicine* from 2002 edition.

### Search Strategy

All the literature was retrieved in WOS on September 2, 2021. The search terms were TS = (osteonecrosis of femoral head OR necrosis of femoral head OR femoral head necrosis OR femoral head necrosis) AND LANGUAGE: (English) AND DOCUMENT TYPES: (Article OR Review). For the time span, we chose the 30 years between 1991 and 2020.

### Data Collection

Full records and cited references were extracted from the retrieved literature for bibliometric analysis, such as titles, years of publications, authors, nationalities, institutions of authors, funding sources, journals of publications, abstracts, keywords, total number of publications, sum times of cited, average citations per item and H-index. The information based on bibliometric characteristics was downloaded from WOS and imported into Microsoft Excel 2017 and VOSviewer (v.1.6.17) for analysis.

### Bibliometric Analysis

Microsoft Excel 2017 was used for statistical analysis and graphing of all the bar charts in the study. In addition, SPSS (v.26.0, IBM, New York, USA) was applied to perform linear regression analysis on the trends of total publications in the last 30 years. Any p values *P* < 0.05 were considered statistically significant. *R* ^2^ represents the degree to which the linear regression model explains the overall variance (19).

VOSviewer is a software for plotting maps based on network data. In the network visualization, items are represented by circles. The size of the circle is determined by the number of publications of the item. The distance between two circles approximately indicates the relatedness of the items. The color of an item is determined by the cluster to which the item belongs. In this study, VOSviewer was used for conducting bibliographic coupling analysis, co-authorship analysis, co-citation analysis and co-occurrence analysis.

## Results and Discussion

### Global Trends of Publications in ONFH

**Figure 1:**
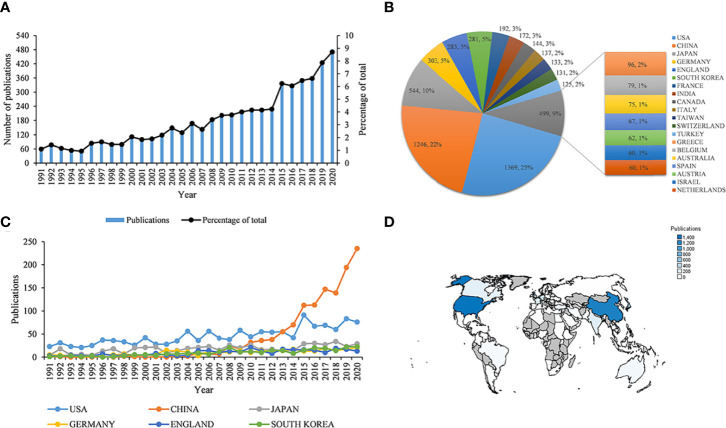
Global trends of publications on osteonecrosis of the femoral head (ONFH) in the last 30 years. **(A)** Annual ONFH-related publications worldwide. **(B)** The total number and percentage of ONFH-related publications from the top twenty countries/regions. **(C)** ONFH-related publications of the top six countries/regions over time. **(D)** Heatmap displaying the distribution of ONFH-related publications globally.

A total of 5,523 ONFH-related literature published between 1991 and 2020 were identified in our study. The total publications of ONFH generally increased over time (*R* ^2^ = 0.872, *P* < 0.001). The annual publications regarding ONFH have grown nearly sevenfold over the three decades from 61 in 1991 to 481 in 2020 (**Figure 1A**). A total of 98 countries/regions published ONFH-related literature, with the top twenty countries/regions shown in **Figure 1B**. Among them, the United States published the most literature (1,369, 24.787%), followed by China (1,246, 22.560%), Japan (544, 9.850%), Germany (303, 5.486%), England (283, 5.124%) and South Korea (281, 5.088%). The United States and China published more than twice as many articles as Japan. Moreover, the total number of ONFH publications in each country or region also increased over time: the United States (*R* ^2^ = 0.732, *P* < 0.001), China (*R* ^2^ = 0.661, *P* < 0.001), Japan (*R* ^2^ = 0.525, *P* < 0.001), Germany (*R* ^2^ = 0.755, *P* < 0.001), England (*R* ^2^ = 0.679, P < 0.001) and South Korea (*R* ^2^ = 0.805, *P* < 0.001) (**Figure 1C**). **Figure 1D** shows the number of publications from individual countries or regions visually in the heatmap.

Bibliometric analysis could be used to evaluate the current status and forecast future directions (20). Therefore, our study was conducted to evaluate and summarize the global trends and current status of ONFH in the last 30 years. As demonstrated in this study, the number of ONFH-related publications has increased significantly over the past three decades from 1991 to 2020. We could predict that ONFH-related publications would continue to increase over time in the coming years. It was easy to conclude from the heatmap that researchers from all over the world were involved in ONFH research, particularly in North America, East Asia and Europe. Highly developed economies and the prevalence of ONFH in these regions may explain this result. China’s contribution was lower than that of the United States before 2013 and gradually surpassed the United States after 2013, even though the total number of publications of the United States in the last 30 years was still more than that of China. This reasonable explanation might be attributed to the rapid national development and the corresponding annually increased research funding for ONFH.

### Quality Analysis of Global Publications

#### Country

**Figure 2:**
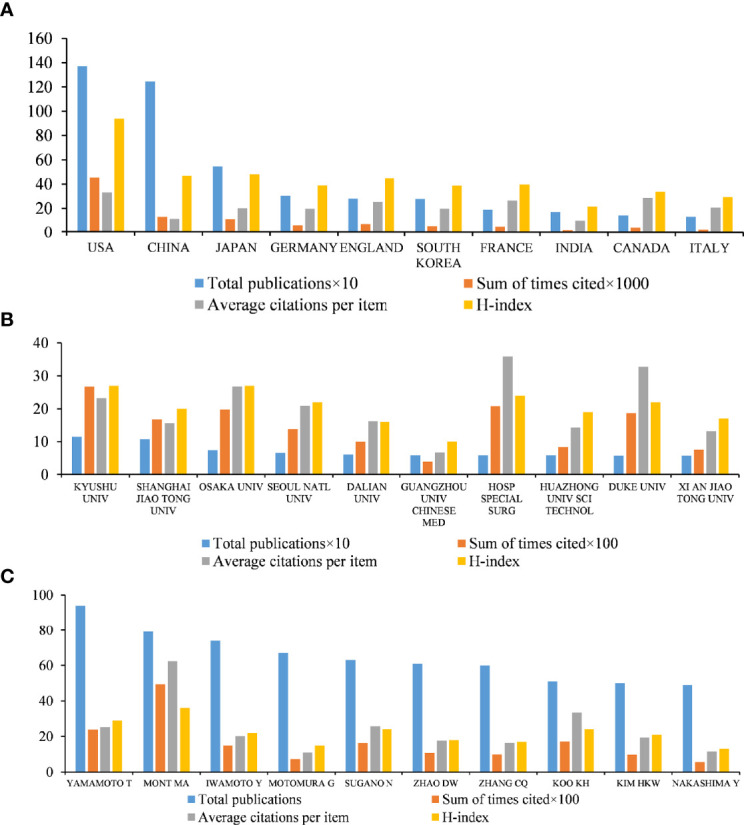
Quality analysis of global publications in ONFH in the last 30 years. **(A)** Total publications, sum of times cited, average citations per item, and H-index of the top ten countries by contributions. **(B)** Total publications, sum of times cited, average citations per item, and H-index of the top ten institutions by contributions. **(C)** Total publications, sum of times cited, average citations per item, and H-index of the top ten authors by contributions.

**Figure 2A** illustrates the sum of times cited, average citations per item and H-index of the top ten countries with the most publications associated with ONFH. Among the top ten countries, the sum of times cited (45,314), average citations per item (33.1) and H-index (94) of the United States, the first-ranked country regarding total publications, were all higher than those of the other nine countries. In terms of the total publications, China ranked second. However, China ranked ninth in average citations per item (11.33), just above India. Other developed countries, including Japan, Germany, England, South Korea, France, Canada and Italy, all had relatively high average citations per item and H-index despite the small number of publications.

In terms of total publications, the sum of times cited, average citations per item and H-index, the United States ranked first in the world and is the leading country in ONFH. China was in second place by the number of total publications and the sum of times cited, ranking third in the H-index, while it only surpassed India in average citations per item among the top ten countries. This means that while China held a leading position in the contributions of ONFH, there was still a significant gap between China and developed countries with regard to the academic level of ONFH research. This situation may be caused by the scientific evaluation system of China favoring the number rather than the quality of publications in the past.

#### Institution

Over the past three decades, approximately 3,721 institutions worldwide have published ONFH-related literature. **Figure 2B** details the top ten most contributing institutions all over the world. Of the ten institutions, five were in China (Shanghai Jiao Tong University, Dalian University, Guangzhou University of Chinese Medicine, Huazhong University of Science and Technology and Xi’an Jiaotong University), two were in Japan (Kyushu University and Osaka University), two were in the United States (Hospital for Special Surgery and Duke University) and one was in South Korea (Seoul National University). The institution making the greatest contribution to ONFH research is Kyushu University, with 115 publications, 2,675 citations, 23.26 average citations and an H-index of 27. Shanghai Jiao Tong University came in second place (107 publications, 1,676 citations, 15.66 average citations and H-index of 20), followed by Osaka University (74 publications, 1,982 citations, 26.78 average citations and H-index of 27), Seoul National University (66 publications, 1,381 citations, 20.92 average citations and H-index of 22) and Dalian University (61 publications, 992 citations, 16.26 average citations and H-index of 16). Interestingly, the top ten most contributing institutions were all from the top ten countries, indicating that the establishment of outstanding institutions was the prerequisite to improve the academic level of a country.

#### Author

Analyzing the quality of publications by the author, the top ten contributors to ONFH are presented in **Figure 2C**. Of the ten authors, five were from Japan (Takuaki Yamamoto, Yukihide Iwamoto, Goro Motomura, Nobuhiko Sugano and Yasuharu Nakashima), two were from China (Dewei Zhao and Changqing Zhang), two were from the United States (Michael A. Mont and Harry K. W. Kim) and the remaining author was from South Korea (Kyung-Hoi Koo). Moreover, eight of these authors were from the top ten institutions: four were from Kyushu University (Takuaki Yamamoto, Yukihide Iwamoto, Goro Motomura and Yasuharu Nakashima), Nobuhiko Sugano was from Osaka University, Dewei Zhao was from Dalian University, Changqing Zhang was from Shanghai Jiao Tong University and Kyung-Hoi Koo was from Seoul National University. The greatest contributor was Takuaki Yamamoto, with 94 publications, 2,379 citations, 25.31 average citations and an H-index of 29. Michael A. Mont, who ranked second in publications, had the greatest number of citations, average citations and highest H-index among the top ten authors (79 publications, 4,927 citations, 62.37 average citations and H-index of 36).

In other words, the top ten authors who contributed most to ONFH were also from the top ten countries and eight of them worked for the top ten institutions. This interesting finding suggested a win–win situation between excellent scientific platforms and first-class scholars. The excellent scientific platforms provided sufficient scientific funds and advanced experimental equipment for top scholars, who were likely to make great contributions to the advancement of such platforms.

### Quality Analysis of Journal and Funding Agency

#### Journal

**Figure 3:**
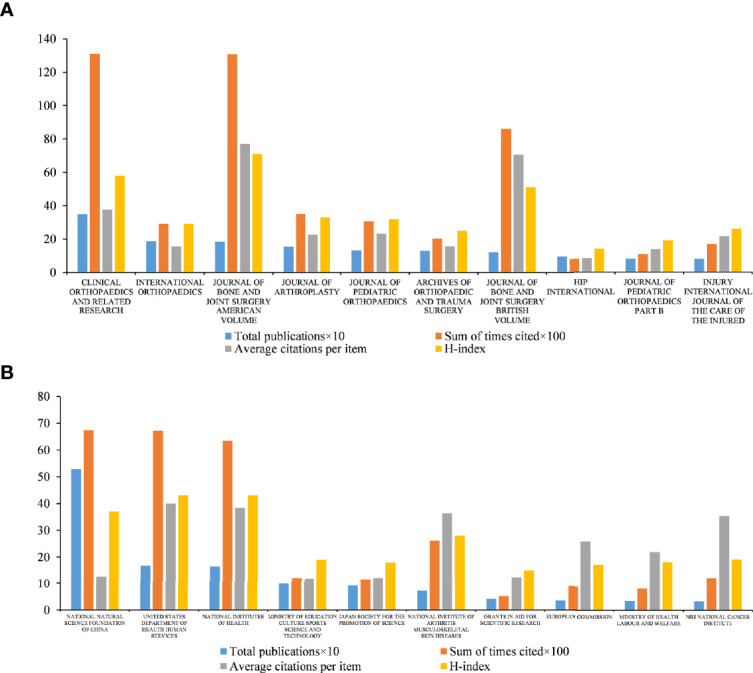
Analysis of highly contributing journals and funding agencies in ONFH in the last 30 years. **(A)** Total publications, sum of times cited, average citations per item, and H-index of the top ten journals. **(B)** Total publications, sum of times cited, average citations per item, and H-index of the top ten funding agencies.

The top ten journals publishing the most ONFH-related literature are shown in **Figure 3A**. *Clinical Orthopaedics and Related Research* (IF = 4.291, 2020) was the most active journal in ONFH research, with 348 articles, followed by *International Orthopaedics* (IF = 3.075, 2020), with 186 articles; *Journal of Bone and Joint Surgery-American Volume* (IF = 5.284, 2020), with 183 articles; *Journal of Arthroplasty* (IF = 4.757, 2020), with 154 articles; *Journal of Pediatric Orthopaedics* (IF = 2.324, 2020), with 132 articles; and *Archives of Orthopaedic and Trauma Surgery* (IF = 3.067, 2020), with 129 articles. From the top ten journals, we could track the latest advances in ONFH by monitoring the latest research of these journals.

Among the top ten journals, *Clinical Orthopaedics and Related Research* published the most ONFH-related articles, nearly twice as many as the publications of the second-ranked journal, *International Orthopaedics*. Additionally, *Clinical Orthopaedics and Related Research* (13,092 citations, 37.62 average citations and H-index of 58) had the most total citations, although the average citations per item were relatively low among the top ten journals. In contrast, the third-ranked journal, *Journal of Bone and Joint Surgery-American Volume*, had the highest average citations and H-index (13,072 citations, 76.9 average citations and H-index of 71). In addition*, Journal of Bone and Joint Surgery-British Volume* had relatively high average citations per item and H-index (8,602 citations, 70.51 average citations and H-index of 51). The higher average citations per item and H-index to some extent indicated the high quality of their publications.

#### Funding Agency

**Figure 3B** presents the top ten funding agencies with the most ONFH-related articles. Globally, the National Natural Science Foundation of China (NSFC, China) funded the most articles with the maximum number of citations (528 publications, 6,739 citations, 12.69 average citations and H-index of 37). The United States Department of Health Human Services (HHS, the United States) ranked second (168 publications, 6,712 citations, 39.95 average citations and H-index of 43), followed by the National Institutes of Health (NIH, the United States) (165 publications, 6,338 citations, 38.41 average citations and H-index of 43), Ministry of Education Culture Sports Science and Technology (MEXT, Japan) (99 publications, 1,216 citations, 11.92 average citations and H-index of 19), Japan Society for the Promotion of Science (JSPS, Japan) (92 publications, 1,156 citations, 12.17 average citations and H-index of 18) and National Institute of Arthritis Musculoskeletal Skin Diseases (NIAMS, the United States) (72 publications, 2,613 citations, 36.29 average citations and H-index of 28). Thanks to the substantial funding from NSFC, China ranked second in terms of the number of ONFH-related publications.

### Top Ten Most Cited Publications in ONFH

**Table 1** shows the top ten most cited publications in ONFH. The top ten publications were published from 1992 to 2012 and the number of citations ranged from 372 to 892. The second, fourth and seventh most cited publications were written by Mont et al., Mankin, and Assouline-Dayan et al. (22, 24, 27). They all comprehensively summarized the etiology, pathogenesis, pathology, diagnosis and treatment of ONFH. Moreover, the ninth most cited publication was written by Steinberg et al., which introduced the Steinberg classification system for ONFH diagnosis, staging and treatment (29). Other literature in the top ten most cited publications involved other topics related to ONFH, such as hip surgery, fracture and trauma. In addition, the vast majority of the top ten most cited publications were published before 2010 and only one article was published in 2012, which was to be expected, as recently published articles take time to be widely cited.

**Table 1:** Top ten most cited publications in osteonecrosis of femoral head (ONFH) in the world.

Title	Author	Journal	Year	Type	IF	Times cited
Surgical dislocation of the adult hip - A technique with full access to the femoral head and acetabulum without the risk of avascular necrosis	Ganz, R et al. (21)	Journal of Bone and Joint Surgery-British Volume	2001	Article	3.309	892
Non-traumatic avascular necrosis of the femoral head	Mont, MA and Hungerford (22),	Journal of Bone and Joint Surgery-American Volume	1995	Review	5.284	746
Fractures of the acetabulum: Accuracy of reduction and clinical results in patients managed operatively within three weeks after the injury	Matta, JM (23)	Journal of Bone and Joint Surgery-American Volume	1996	Article	5.284	733
Nontraumatic necrosis of bone (osteonecrosis)	Mankin, HJ (24)	New England Journal of Medicine	1992	Review	91.253	600
Anterior femoroacetabular impingement Part II. Midterm results of surgical treatment	Beck, M et al. (25)	Clinical Orthopaedics and Related Research	2004	Article	4.291	511
Biological reactions to wear debris in total joint replacement	Ingham, E et al. (26)	Proceedings of the Institution of Mechanical Engineers. Part H-Journal of Engineering in Medicine	2000	Review	0.740	491
Pathogenesis and natural history of osteonecrosis	Assouline-Dayan, Y et al. (27)	Seminars in Arthritis and Rheumatism	2002	Review	5.532	475
Anatomy of the medial femoral circumflex artery and its surgical implications	Gautier, E et al. (28)	Journal of Bone and Joint Surgery-British Volume	2000	Article	3.309	436
A quantitative system for staging avascular necrosis	Steinberg, ME et al. (29)	Journal of Bone and Joint Surgery-British Volume	1995	Article	3.309	435
Corrosion at the head-neck taper as a cause for adverse local tissue reactions after total hip arthroplasty	Cooper, HJ et al. (30)	Journal of Bone and Joint Surgery-American Volume	2012	Article	5.284	372

### Research Areas Analysis

**Figure 4:**
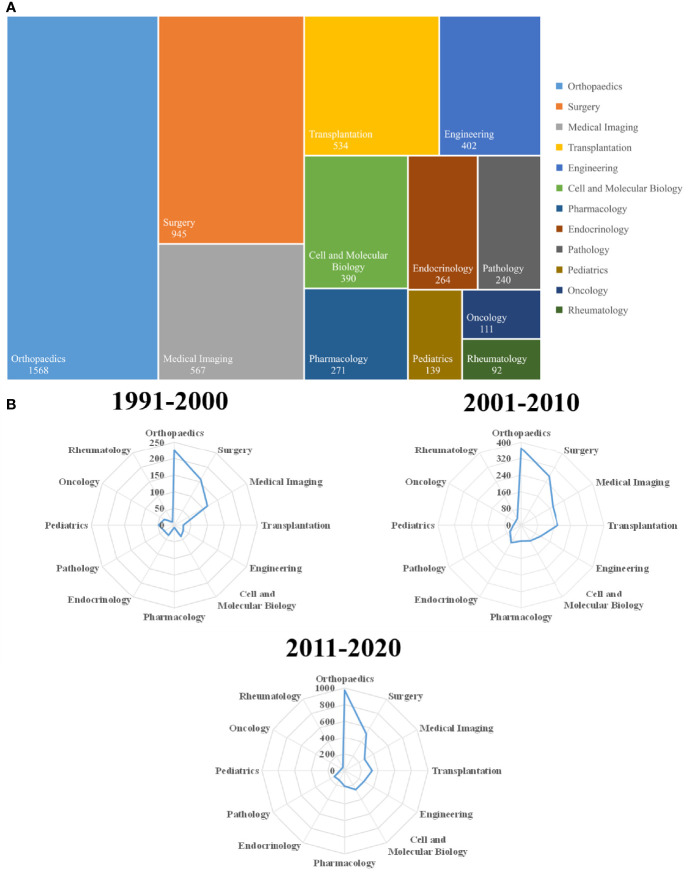
Research area analysis of global publications in ONFH in the last 30 years. **(A)** Research areas in ONFH. **(B)** Radar map of research areas in ONFH during 1991-2000, 2001-2010 and 2011-2020.

The ONFH-related articles published from 1991 to 2020 were categorized into 83 different research areas on WOS. However, to more intuitively show the changes in the research areas over time, we merged the areas of similar significance and finally selected 12 research areas. **Figure 4A** presents all the research areas and the corresponding number of ONFH-related articles, among which the orthopaedics area received the most attention (1,568, 28.390%), followed by surgery (945, 17.110%), medical imaging (567, 10.266%), transplantation (534, 9.669%), engineering (402, 7.279%), cell and molecular biology (390, 7.061%), pharmacology (271, 4.907%), endocrinology (264, 4.780%), pathology (240, 4.345%), pediatrics (139, 2.571%), oncology (111, 2.010%) and rheumatology (92, 1.666%). **Figure 4B** shows the distribution of ONFH-related research areas during 1991-2000, 2001-2010 and 2011-2020, from which we could obviously find the changes in ONFH-related research areas. Orthopaedics, surgery and medical imaging have always been the research areas of greatest concern, even though the popularity of medical imaging has decreased to some extent. Over time, research in transplantation, engineering, cell and molecular biology, pharmacology, and endocrinology has increased considerably. Pathology, pediatrics, oncology and rheumatology have not received much attention all the time.

Orthopaedics, surgery and medical imaging have been extensively and intensively studied due to their focus on the risk factors, etiology, diagnosis and treatment of ONFH. With the development of vascularized bone grafts, bone marrow mesenchymal stem cell transplantation and endothelial progenitor cell transplantation, an increasing number of studies have been conducted to test the efficacy and safety of transplant therapy for ONFH through animal experiments or clinical trials. Furthermore, researchers combined transplantation with cell and molecular biology to enhance the efficacy of transplant therapy for ONFH. For example, Hang et al. evaluated the efficacy of vascular endothelial growth factor 165 (VEGF165) transgenic bone marrow mesenchymal stem cells in mongrel dogs with ONFH (31). In the engineering area, novel biomechanical analysis, materials and implants related to ONFH are hotspots in this field. For example, Wang et al. developed a device referred to as the super-elastic cage for the treatment of Ficat and Arlet stage II and III ONFH (32). In addition to surgical treatment, researchers explored drug therapy, which we classified into pharmacology. In recent years, some traditional Chinese medicines have gradually been studied for ONFH treatment, such as Panax notoginseng saponin, bone-strengthening pills and Huogu I formula (33–35). Endocrinology focused on the effects and mechanisms of long-term or excessive GC administration on ONFH. Pediatrics, oncology and rheumatology mainly studied the role of Legg-Calvé-Perthes disease, leukemia and systemic lupus erythematosus in the pathogenesis of ONFH, respectively. Legg-Calvé-Perthes disease is the most prevailing juvenile form of idiopathic ONFH, affecting children 2-14 years old (36). ONFH is one of the most common and debilitating therapy-related side effects of antileukemic treatment and is associated with the treatment of GCs, asparaginase (ASP) and methotrexate (MTX), genetic factors and age (37). Similarly, ONFH is also one of the serious and well-recognized complications of SLE and the use of GCs, an impaired immune microenvironment and the complex pathogenesis of SLE are synergistically involved in the pathogenesis of ONFH (38).

### Bibliographic Coupling Analysis

Bibliographic coupling analysis is a method exhibiting the relatedness of items based on the number of references they share, which is established when two items cited the same article. Bibliographic coupling analysis was used in this study to establish the similarity relationship among publications from the three dimensions of country, institution and journal. To some extent, the total link strength of a particular item can explain its worldwide influence. Items with higher total link strength indicate that the countries/institutions/authors are more globally influential.

#### Country

**Figure 5:**
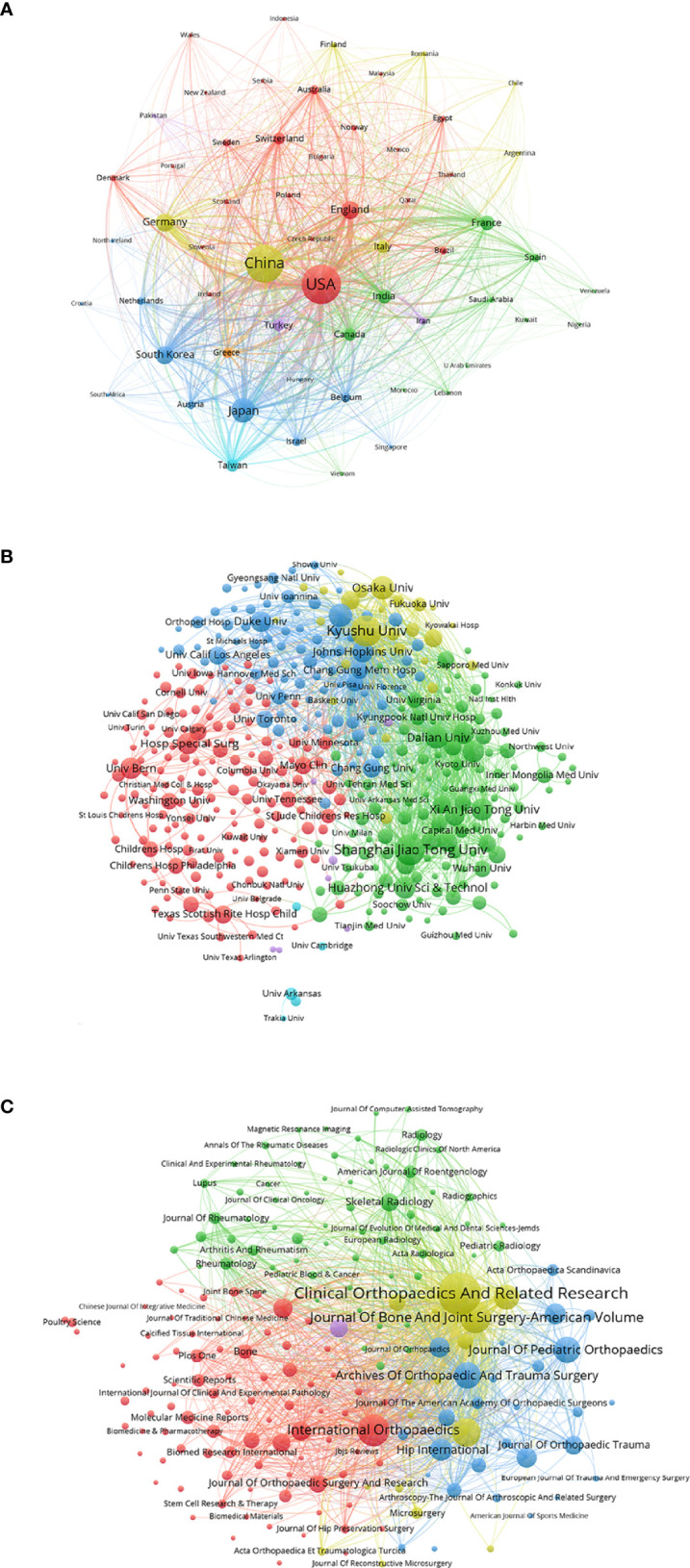
Bibliographic coupling analysis of global publications in ONFH in the last 30 years. **(A)** Network visualization of the 59 identified countries in ONFH. **(B)** Network visualization of the 405 identified institutions in ONFH. **(C)** Network visualization of the 189 identified journals in ONFH. In the visualized network, each item is represented by a circle. The size of the circle is determined by the number of publications of the item. The distance between two circles approximately indicates the relatedness of the items. The color of an item is determined by the cluster to which the item belongs.

**Figure 5A** shows the relationship of 59 identified countries (the minimum number of documents of a country is over five) in total link strength using VOSviewer. The top six countries by total link strength were as follows: the United States (total link strength = 1,066,241 times), China (total link strength = 737,151 times), Japan (total link strength = 427,254 times), South Korea (total link strength = 280,129 times), Germany (total link strength = 273,705 times) and England (total link strength = 214,591 times). Therefore, the United States was the leading country in ONFH worldwide according to bibliographic coupling analysis, corresponding to the highest total publications, sum of times cited, average citations per item and H-index of the United States in ONFH described earlier.

#### Institution

**Figure 5B** details the relationship of 405 identified institutions (the minimum number of documents of an institution is over five) in total link strength using VOSviewer. The top six institutions by total link strength were as follows: Kyushu University (total link strength = 137,084 times), Johns Hopkins University (total link strength = 130,922 times), Sinai Hospital (total link strength = 108,755 times), Shanghai Jiao Tong University (total link strength = 94,142 times), Dalian University (total link strength = 91,473 times) and Seoul National University (total link strength = 84,178 times). Therefore, Kyushu University was the leading institution in ONFH globally according to bibliographic coupling analysis, corresponding to the highest total publications, sum of times cited and H-index of Kyushu University in ONFH introduced above.

#### Journal

**Figure 5C** presents the relationship of 189 identified journals (the minimum number of documents of a journal is over five) in total link strength using VOSviewer. The top six journals by total link strength were as follows: *Clinical Orthopaedics and Related Research* (total link strength = 308,010 times), *Journal of Bone and Joint Surgery-American Volume* (total link strength = 208,262 times), *International Orthopaedics* (total link strength = 135,281 times), *Journal of Arthroplasty* (total link strength = 132,289 times), *Journal of Bone and Joint Surgery-British Volume* (total link strength = 119,992 times) and *Archives of Orthopaedics and Trauma Surgery* (total link strength = 96,174 times). Therefore, *Clinical Orthopaedics and Related Research* was the leading journal in ONFH globally according to bibliographic coupling analysis, corresponding to the highest total publications and sum of times cited of *Clinical Orthopaedics and Related Research* in ONFH presented earlier.

### Co-Authorship Analysis

Co-authorship analysis is a measure to determine the connectivity of items based on the number of co-authored publications. Co-authorship analysis was used to evaluate the cooperation between items in our study by counting the number of co-authored publications. In a way, the total link strength of a particular item can reflect the extent to which they are willing to cooperate with others. Items with higher total link strength indicate that the countries/institutions/authors are more willing to collaborate with others.

#### Country

**Figure 6:**
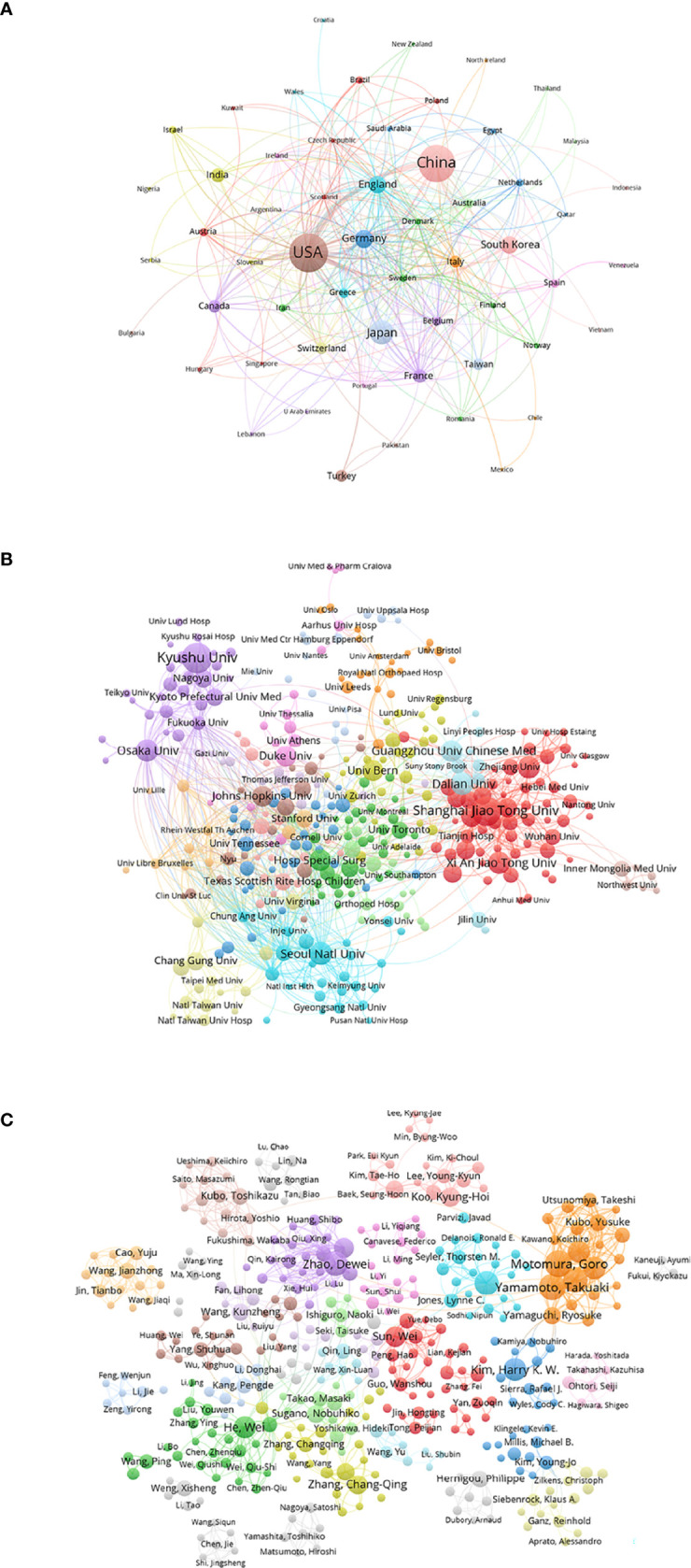
Co-authorship analysis of global publications in ONFH in the last 30 years. **(A)** Network visualization of the 59 identified countries in ONFH. **(B)** Network visualization of the 405 identified institutions in ONFH. **(C)** Network visualization of the 629 identified authors in ONFH. In the visualized network, each item is represented by a circle. The size of the circle is determined by the number of publications of the item. The distance between two circles approximately indicates the relatedness of the items. Item color is determined by the cluster to which the item belongs.

**Figure 6A** shows the relationship of 59 identified countries (the minimum number of documents of a country is over five) in total link strength using VOSviewer. The top six countries by total link strength were as follows: the United States (total link strength = 353 times), England (total link strength = 179 times), Germany (total link strength = 149 times), China (total link strength = 144 times), France (total link strength = 112 times) and Italy (total link strength = 86 times). Therefore, authors from the United States were more cooperative than those from other countries according to co-authorship analysis.

#### Institution

**Figure 6B** details the relationship of 405 identified institutions (the minimum number of documents of an institution is over five) in total link strength using VOSviewer. The top six institutions by total link strength were as follows: Seoul National University (total link strength = 125 times), Kyungpook National University (total link strength = 104 times), Stanford University (total link strength = 97 times), Osaka University (total link strength = 95 times), Fukuoka University (total link strength = 91 times) and John Hopkins University (total link strength = 88 times). Therefore, Seoul National University was more cooperative than the others according to co-authorship analysis.

#### Author

**Figure 6C** exhibits the relationship of 629 identified authors (the minimum number of documents of an author is over five) in total link strength using VOSviewer. The top six authors by total link strength were as follows: Goro Motomura (total link strength = 328 times), Takuaki Yamamoto (total link strength = 272 times), Satoshi Ikemura (total link strength = 226 times), Yukihide Iwamoto (total link strength = 272 times), Yasuharu Nakashima (total link strength = 218 times) and Dewei Zhao (total link strength = 186 times). Therefore, Goro Motomura was the most cooperative author according to co-authorship analysis.

### Co-Citation Analysis

Co-citation analysis refers to a method presenting the relatedness of items based upon the number of times they are cited together, which is established when two items are both cited in another article. Compared with bibliographic coupling analysis, co-citation analysis can more scientifically highlight the influence of items. Items with higher total link strength indicate that the journals/publications are more influential globally.

#### Journal

**Figure 7:**
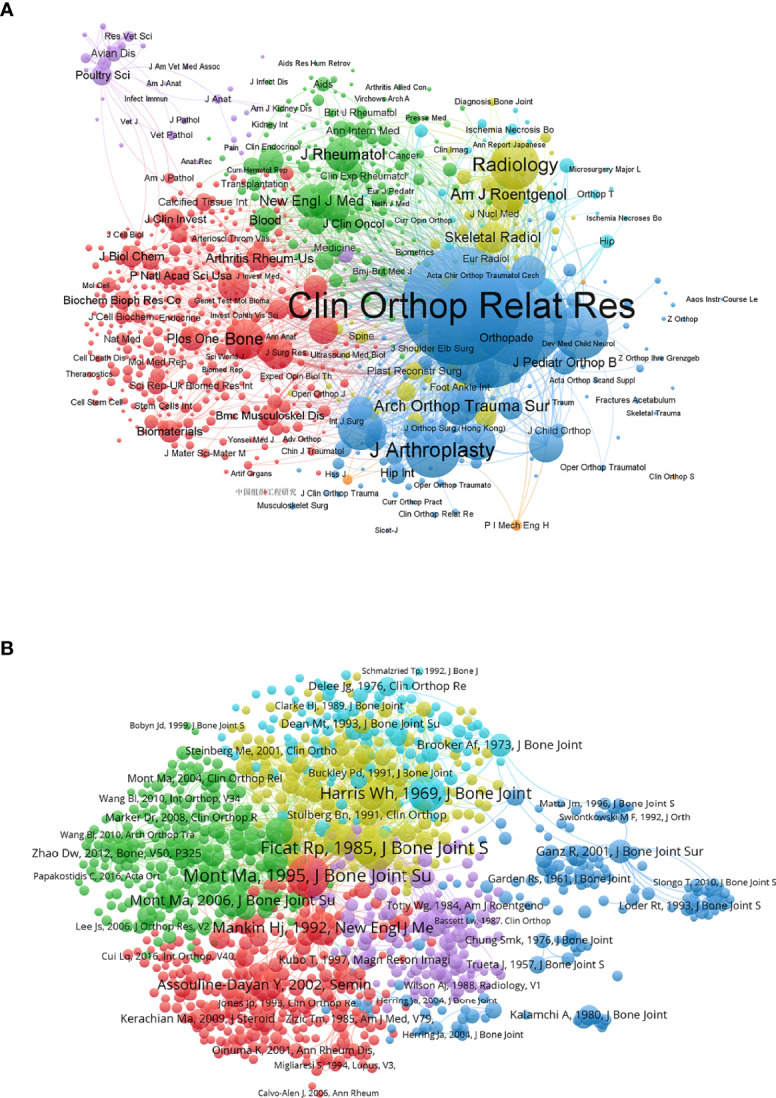
Co-citation analysis of global publications in ONFH in the last 30 years. **(A)** Network visualization of the 841 identified journals in ONFH. **(B)** Network visualization of the 1,184 identified publications in ONFH. In the visualized network, each item is represented by a circle. The size of the circle is determined by the number of publications of the item. The distance between two circles approximately indicates the relatedness of the items. Item color is determined by the cluster to which the item belongs.

**Figure 7A** displays the relationship of 841 identified journals (the minimum number of citations of a journal is over twenty) in total link strength using VOSviewer. The top six journals by total link strength were as follows: *Clinical Orthopaedics and Related Research* (total link strength = 625,944 times), *Journal of Bone and Joint Surgery-American Volume* (total link strength = 582,344 times), *Journal of Bone and Joint Surgery-British Volume* (total link strength = 415,827 times), *Journal of Arthroplasty* (total link strength = 162,360 times), *Journal of Pediatric Orthopaedics* (total link strength = 125,262 times) and *Radiology* (total link strength = 114,859 times). Therefore, *Clinical Orthopaedics and Related Research* was the predominant journal in ONFH globally according to co-citation analysis.

#### Publication

**Figure 7B** reveals the relationship of 1,184 identified publications (the minimum number of citations of a publication is over twenty) in total link strength using VOSviewer. The top six publications by total link strength were as follows: Mont et al. (22) (total link strength = 10,027 times), Ficat (39) (total link strength = 9,686 times), Steinberg et al. (29) (total link strength = 7,134 times), Harris (40) (total link strength = 6,067 times), Mont et al. (41) (total link strength = 5,983 times) and Mankin (24) (total link strength = 5,523 times). Therefore, Mont et al. was the most influential publication in ONFH globally according to co-citation analysis.

### Co-Occurrence Analysis

**Figure 8:**
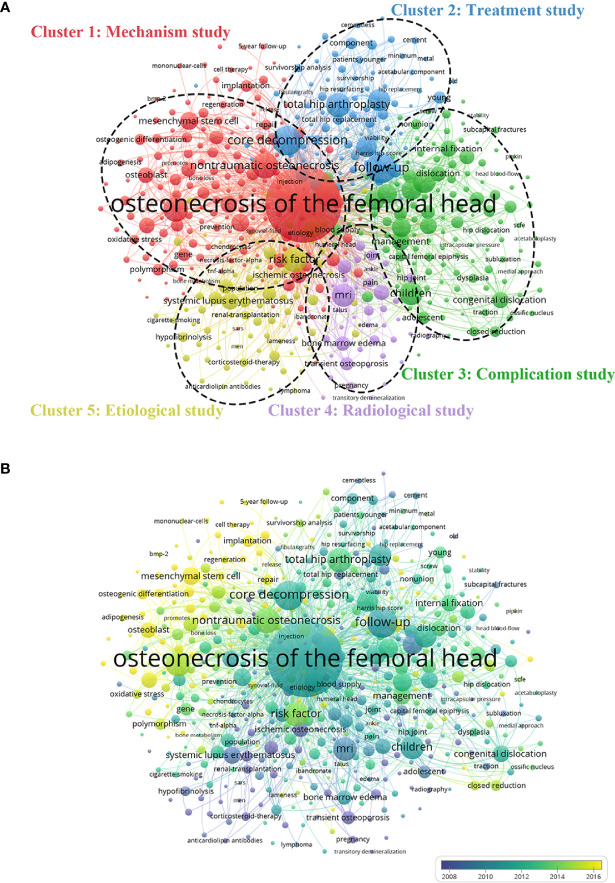
Co-occurrence analysis of global publications in ONFH in the last 30 years. **(A)** Network visualization of 515 identified keywords in ONFH. All the keywords are divided into 5 clusters: “Mechanism study”, “Treatment study”, “Complication study”, “Radiological study” and “Etiological study”. **(B)** Overlay visualization of the 515 identified keywords in ONFH based on the average time they appeared in the publications. The blue keyword appeared earlier, while the yellow keyword appeared later.

The co-occurrence network visualization is created by analyzing the number of articles in which keywords occurred together in titles or abstracts. The aim is to determine the hot research directions and topics critical for tracking the development of science (20). As illustrated in **Figure 8A**, 515 identified keywords (the minimum number of occurrences of a keyword in titles and abstracts is over ten) are classified into five clusters: “Mechanism study”, “Treatment study”, “Complication study”, “Radiological study” and “Etiological study”. In the “Mechanism study” cluster, the most used keywords were nontraumatic osteonecrosis, steroid-induced osteonecrosis of the femoral head, mesenchymal stem cell and apoptosis. For the “Treatment study” cluster, the frequently used keywords were core decompression, follow-up, total hip arthroplasty and replacement. In the “Complication study” cluster, the most used keywords were children, femoral neck fracture, complication and management. For the “Radiological study” cluster, the major keywords were MRI, diagnosis, bone marrow edema and transient osteoporosis. In the “Etiological study” cluster, the main keywords were risk factor, natural history, systemic lupus erythematosus and bone mineral density. Our results suggested that nontraumatic osteonecrosis drew the most attention worldwide, which was consistent with the reality that long-term or excessive GC administration was the main pathogenesis of ONFH. Beyond that, other keywords with higher total link strength were of great significance in their respective fields. These results could provide novel insights into the hot research directions and topics of ONFH, which indicated the promising fields need further attention and high-quality research in the future.

The overlay visualization is identical to the network visualization except for the colors of items, highlighting their average appearing time. As presented in **Figure 8B**, keywords are colored differently depending on the average time they appear in the publications. The blue keywords appeared earlier, while the yellow keywords appeared later. The results of co-occurrence analysis indicated that “Mechanism study” might become the hot spot of future ONFH research, while before 2010, most studies focused on the “Complication study” and “Radiological study”. Furthermore, several cutting-edge keywords related to the pathogenesis of ONFH, including mesenchymal stem cells, apoptosis, oxidative stress, adipogenesis, osteogenic differentiation and endothelial progenitor cells, appeared in the overlay visualization (11, 42–45). Recent studies indicated that GCs played a significant role in the shift between adipogenic and osteogenic differentiation of mesenchymal stem cells *via* Runx2, TAZ, PPARγ and C/EBP signaling pathways, which was a pivotal pathogenesis of ONFH (46). In addition, oxidative stress is also involved in regulating the adipogenesis and osteogenesis of mesenchymal stem cells to influence the progression of ONFH (46). Endothelial progenitor cells play an essential role in maintaining the normal structure and function of bone vessels, and the impaired angiogenesis, thrombosis and fat embolism caused by abnormal apoptosis and dysfunction of bone endothelial cells are involved in the pathogenesis of ONFH (47). Therefore, these directions deserve more time and funds for more in-depth and comprehensive research in the future. Moreover, our research results are expected to provide some theoretical basis for several countries or funding agencies to make more scientific and reasonable investment plans or talent introduction plans.

### Strengths and Limitations

Our study provided novel insight into the global trends and current status of ONFH-related publications by using bibliometric methods. However, there are some limitations to the study that must be acknowledged. First, bibliometric analysis measures the quality of publications mainly by comparing the sum of times cited and average citations per item. However, there is no absolute equivalence between them. In other words, highly cited publications are not equivalent to high scientific quality. Second, considering that WOS is one of the most commonly used databases worldwide and is convenient for bibliometric analysis, we chose WOS as our only database for literature retrieval. Due to the differences in the publications covered by the major databases, including WOS, PubMed, and the Cochrane Library, we may have omitted several publications from the analysis, leading to database bias. Third, we only analyzed publications written in English and excluded non-English publications, which might result in language bias. Moreover, we did not screen the 5,523 publications to ensure that they were relevant to ONFH because we limited the document type to articles or reviews, which might lead to some error in the accuracy of our results. We made this choice because we limited the document type to articles or reviews and excluded books, corrections, letters, meeting abstracts, meeting summaries and retractions in the search terms to avoid retrieving publications irrelevant to ONFH from the WOS database. Finally, the effect of publication time on the sum of times cited was not considered. Several of the latest articles with high quality may not attract our attention because of the low sum of times cited. Therefore, it is also essential to pay attention to the latest publications.

## Conclusion

This study identified ONFH-related publications in the last 30 years and presented their global trends and current status. The total publications of ONFH generally increased over time in the last three decades. The United States consistently ranked first in total publications, sum of times cited, average citations per item and H-index. Kyushu University, Osaka University, Hospital for Special Surgery and Duke University were the main contributing institutions to ONFH. *Clinical Orthopaedics and Related Research*, *Journal of Bone and Joint Surgery-American Volume* and *Journal of Bone and Joint Surgery-British Volume* were the major publishing channels for ONFH-related articles. Takuaki Yamamoto and Michael A. Mont were the main contributors to ONFH. Transplantation, engineering, cell and molecular biology, pharmacology and endocrinology have gradually increased and become hot topics in ONFH research. Furthermore, mechanism study in ONFH including mesenchymal stem cells, apoptosis, oxidative stress, adipogenesis, osteogenic differentiation and endothelial progenitor cells, have attracted more attention and will become a hot spot in the future.

## Data Availability Statement

The original contributions presented in the study are included in the article/**Supplementary Material**. Further inquiries can be directed to the corresponding authors.

## Author Contributions

RD, CH, and WX conceived the study. ZW, YL, ZC, MF, and JW participated in statistical analysis. ZW wrote the manuscript. All authors contributed to the article and approved the submitted version.

## Funding

This work was supported by National Key R&D Program of China (2019YFA0111900), National Natural Science Foundation of China (No. 81874030, 81902203, 82072506), Hunan Young Talents of Science and Technology (No.2021RC3025) and Elite Medical Professionals Project of China-Japan Friendship Hospital (ZRJY2021-QM21).

## Conflict of Interest

The authors declare that the research was conducted in the absence of any commercial or financial relationships that could be construed as a potential conflict of interest.

## Publisher’s Note

All claims expressed in this article are solely those of the authors and do not necessarily represent those of their affiliated organizations, or those of the publisher, the editors and the reviewers. Any product that may be evaluated in this article, or claim that may be made by its manufacturer, is not guaranteed or endorsed by the publisher.
